# Supplementary material for: Genetic and Phenotypic Evaluation of European Maize Landraces as a Tool for Conservation and Valorization of Agrobiodiversity
Source: Biology (Basel). 2024 Jun 19;13(6):454. doi: 10.3390/biology13060454 (PMC11201045; doi:10.3390/biology13060454)
Supplement: Supplementary file 1 [file biology-13-00454-s001.zip › biology-2990264-supplementary.pdf]

SUPPLEMENTARY FILE for Balconi et al., Genetic and phenotypic evaluation of European maize landraces for conservation and valorization of agrobiodiversity.

Content:

Supplementary Information S1: Description of Genebanks involved in the EVA Maize network.

Supplemental Figures:

Figure S1: Genetic diversity (Hs) frequency for the complete panel and genetic groups from ADMIXTURE (K=9).

Figure S2: Determination of the K value for ADMIXTURE using deltaK.

Figure S3: Ward dendrogram of genetic diversity based on Modified Rogers Distance of 626 landraces, for different K values.

Figure S4: Neighbour joining dendrogram based on F_{st} between 626 landraces.

Figure S5: Principal Coordinate Analysis based on Modified Roger's Distance estimated with 23,412 SNP, representing i) axis 1 and axis 3, ii) axis 1 and axis 4.

Figure S6: Map of origins of 483 EVA Maize accessions colored according to their belonging to genetic groups.

Figure S7: Relationship for female flowering (days to silking, DS) and plant height (PH) among 588 maize landraces of the EVA collection.

Supplementary Information S1:

Description of Genebanks involved in the EVA Maize network
The EVA Maize Network (https://www.ecpgr.cgiar.org/eva/eva-networks/maize) involves research institutions, genebanks and private breeding companies from nine European countries: Croatia, France, Germany, Italy, Portugal, Romania, Serbia, Spain and Switzerland. Below follow short descriptions of the participating genebanks and their maize collections.

Croatia: University of Zagreb, Faculty of Agriculture (HRV041)
The *ex situ* collection of maize in the **Croatian Genebank** includes 88 inbred lines and 170 landraces, most of which are kept in the collection of the **University of Zagreb, Faculty of Agriculture** (https://www.agr.unizg.hr/). The inbred lines have different proportions of maize landraces from Southeastern Europe in their pedigrees. The landraces were collected recently (from 2007 to 2019) on the territory of the Republic of Croatia.

France: INRAE – Montpellier (FRA015)
The collection of maize populations of the French **National Research Institute for Agriculture, Food and the Environment (INRAE)** (https://www.inrae.fr/) is conserved *ex situ* and managed by the **Biological Resource Center GAMéT of AGAP Institut** (Univ Montpellier, CIRAD, INRAE, Institut Agro) located in Montpellier at the ARCAD building (https://doi.org/10.18167/infrastructure/00007»). The collection of populations is managed in a network including the INRAE research units and the French maize breeding compagnies belonging to the Promaïs association (http://pro-maize-corn.com/). The maize populations collection conserved in BRC GAMéT (Montpellier, France) includes the French National collection with 465 maize populations declared in TIRPAA (265 landraces of metropolitan France, 108 landraces of Guadeloupe and synthetics and composites) and the network population collection. Another 1,600 populations not part of the national collection are conserved *ex situ* in the cold room of the ARCAD building.

Italy: CREA-CI Bergamo (ITA386)
The **Consiglio per la Ricerca in Agricoltura e l'Analisi dell'Economia Agraria (CREA)** is a National Research Organization of the Italian Ministero dell'Agricoltura, della Sovranità Alimentare e delle Foreste (MASAF), with scientific competence within agriculture, agroindustry, economics, food, fishery and forestry (https://www.crea.gov.it/en/home). CREA is the leading Italian research organization dedicated to agri-food supply chains. The CREA Research Centre for Cereal and Industrial Crops (CREA-CI) includes seven territorial sites focused on quality and yield crop improvement through multidisciplinary approaches. The experimental maize farming station in Bergamo was established in the 1950's. The focus of the institute is on the in-depth study of several aspects around maize, such as breeding, chemistry, pathology and genetics. When in Italy hybrids were introduced from USA, landraces from agricultural consortia spread across the national territory were collected in the Genebank of CREA-CI Bergamo to preserve biodiversity and genetic resources *ex situ*. The Italian maize germplasm collection is one of the largest, both for original contributions and for local diversity in the Southern Europe climatic zone that had favoured the diversification. The wide range of pedoclimatic situations in Italy and different cultivation practices originated a large amount of local varieties (landraces), promoting selection of flint and semi-flint maize, with a bigger yield in milling and a particular suitability in food flour production (Revilla et al., 2022). Currently, the maize Genebank of CREA-CI Bergamo holds more than 3,000 inbred lines from Italy and foreign countries, mutants and synthetic accessions and 1,295 landraces of which around 720 are from Italy, while the rest come from 20 different countries.

Portugal: INIAV-BPGV, Braga (PRT001)
The **Portuguese Genebank (Banco Português de Germoplasma Vegetal, BPGV,** https://iniav.pt/bpgv**)** began the regular collecting and conservation of maize genetic resources (*Zea mays* L.) in 1977 with technical and financial support from the Food and Agriculture Organism of the United Nations (FAO) and from Bioversity International/IBPGR. In 1978, Dr. Erna Benett, then coordinator of the IBPGR genetic resources program for the Mediterranean region, proposed that BPGV conserve all the duplicates of maize accessions from the

Mediterranean basin countries as a base collection. In 1983, BPGV became part of the IBPGR global network of designed security base collection centers, and is responsible for conserving safety duplicates of maize collections from countries across the Mediterranean region. The Portuguese maize collection consists of 12,098 accessions of *Zea mays* from different sources and countries; among the accessions from Portugal, 2,091 accessions are landraces, which were collected in all representative regions of Portugal, including the Azores and Madeira islands.
The evaluation of the collection began in 1981, with characterization based on descriptors used at an international level (IPGRI). Evaluations are performed continuously since then, to date 1,659 accessions have been evaluated.

Portugal: ESAC-IPC, Coimbra (PRT053)
The **Escola Superior Agraria de Coimbra** (ESAC-IPC https://www.esac.pt//) has been able throughout the years to collect germplasm landraces, characterize them and share it among the farmers, continuing the work that was started by Silas Pêgo in Participatory Plant Breeding in 1984. IPC germplasm collecting missions are shared with BPGV for germplasm long term conservation.
The collected material is studied in trials with farmer involvement. Multiplication, selfing, crosses and breeding for populations is ongoing in our nursery. The ESAC has 140 ha of experimental fields and from these 12 ha are devoted to organic production, where breeding and selection for organic farming are being done. Main activities at ESAC are focused on phenotyping, facilities include a cold chamber and tools such as a seed counter and equipment for ears and kernels characterization.

Romania: Suceava Genebank (ROM007)
The **Bănca de Resurse Genetice Vegetale Suceava,** (https://svgenebank.ro/), Romania's national genebank, was established in 1990 as an autonomous institution of national importance, in the present being subordinated to the Academy of Agricultural and Forest „Gheorghe Ionescu Șișești", Bucharest.
The main objectives of the genebank are: to extend the genetic diversity of the Gene bank's collections (seeds, in vitro and live plants); to keep the national collection (medium and long terms) for all seed propagated crop plants and their wild relatives; to provide germplasm with high level of resistance to biotic and abiotic stress factors to Romanian plant breeding programs; to promote in *situ-on farm* conservation of local varieties and traditional knowledges and provide seeds from Gene bank's collections to people interested in growing traditional cultivars. Activities are focused on the following directions: acquisition of germplasm, multiplication and regeneration of germplasm, germplasm characterization and evaluation, storing of genetic material, data management, distribution of the germplasm, promoting the utilization of the germplasm, sustaining regional and international collaboration participating in ECPGR, coordinating national PGR activities and representing Romania in relevant international organizations. The Suceava Genebank hosts a rich and diverse genetic heritage, its collection is made up of 597 sub-specific taxa, 393 species, 219 genera and 39 botanical families, representing more than 25,000 different varieties (wild forms, local populations, material breeding, etc.), kept as: *ex situ* seeds (over 24,500 varieties); live plants in the experimental field (332 potato and garlic genotypes) and *in vitro* seedlings under slow growth conditions (118 local potato genotypes).

Serbia: Maize Research Institute Zemun Polje (SRB001)
The **Maize Research Institute Zemun Polje** (Institut za kukuruz "Zemun Polje", MRIZP, https://mrizp.rs/) is a Serbian leading public institution for research and development of new high-yielding and quality maize hybrids, soybean cultivars and small grains varieties, adapted to diverse agro-ecological conditions, and suitable for a variety of purposes and uses. The main MRIZP objectives are: (i) to develop the programmes focused on plant breeding, seed production, cultivation, utilisation and market placement of cultivars, varieties and hybrids both in the country and abroad, and (ii) to develop the technological methods and processing procedures for the production of food and feed, and for industrial needs.
The MRIZP Genebank (MRIZPGB) ranks among the ten largest in the world and five largest in Europe. It maintains the collection of 5,806 maize accessions: 2,217 local landraces from the Western Balkan; 1,335 heterozygous accessions (populations, synthetics and composites) and 2,254 inbred lines, introduced from

40 countries worldwide. The accessions are with various kernel type, colour and nutrient content, adapted to temperate climate, with high potential for food production with increased health benefits. The MRIZP genebank collection was screened for identification of sources for cytoplasmic male sterility and herbicide tolerance. Within pre-breeding activities, due to more frequent unpredictable and extreme climatic events, as well as constantly growing demand for healthy food and feed, particular attention has been paid to identification of sources for drought tolerance and improved grain quality. According to agronomic traits of interest and good general combining ability, two mini-core collections were created and included in commercial breeding programmes.

Spain: Misión Biológica de Galicia, Pontevedra (MBG-CSIC), Plant Genetic Resources Center of Spain (CRF-CSIC) (ESP004-007-009-016-119)

The **Plant Genetic Resources Center of Spain** (Centro de Recursos Fitogenéticos y Agricultura Sostenible, CRF, https://www.inia.es/unidades/Institutos%20y%20Centros/CRF/Paginas/Home.aspx) maintains a collection of 2,936 maize accessions from diverse origins. Among those accessions, 37 are unallocated, while other accessions come from diverse specific Spanish regions, Galicia being the region with the highest representation (774), followed by Cantabria (379), the Canary Islands (292), Andalusia (279), the Basque Country (249) and Asturias (181).

The Spanish maize collection begun in 1921 by Cruz Gallastegui, the founder of the **Misión Biológica de Galicia MBG-CSIC** (https://mbg.csic.es/en/the-mision-biologica-de-galicia/). Subsequently, large collections were made, but in the 1940s, Enrique Sánchez Monje grouped those accessions in 52 races based on morphology and genetic relationships, and the original collection was lost. However, since 1974, Amando Ordás, Jesus Moreno-González and collaborators have made large collections of maize accessions that have been maintained and enlarged until today. Nowadays, the Spanish landrace active collection conserved in the germplasm bank of the MBG-CSIC comprises 177 Spanish field corn and 25 Spanish popcorn landraces. A national collection of 90 varieties was constructed based on morphological, agronomic and molecular characterization as well as on previous knowledge of breeders.

Switzerland: Agroscope, Nyon (CHE001)

Agroscope (https://www.agroscope.admin.ch/agroscope/en/home.html) is the Swiss public, nonprofit research institution for all sectors of agriculture. Agroscope is associated with the Swiss Federal Department of Economic Affairs, Education and Research (WBF). Research conducted at Agroscope provides tools and support to enable the production of safe and healthy food for an environmentally respectful agriculture. The conservation of genetic resources in Switzerland began around 1900. At that time, researchers at the Federal agricultural station collected local varieties of wheat and barley, and selected better-performing varieties from these local populations. The collection of maize started in the 1940s. Today Agroscope maintains a collection of 429 maize accessions, including 206 landraces from Switzerland and a few from Liechtenstein and neighboring countries and 223 inbred lines, used in the breeding program. During the Second World War great efforts were made to collect the diversity of cultivated plants and to analyse their suitability for cultivation and breeding. Thus, the research station collected from 1941-1942 more than 100 different maize landraces. The responsible researcher was not only interested in well-known regional varieties such as Rheintaler- and Linthmais, but also in different shapes and colours. As a result, a very colourful palette of maize shapes has been preserved. In a second phase of collecting landraces started at the beginning of the 1960s, a further 43 origins from Graubünden and Valais were added, and in the last 15 years 76 landraces originating from Valais, Ticino, the Rhine Valley and the Linth region were collected. Maize breeding began in Switzerland around 1930. Various landraces were used to develop inbred lines used in classical hybrid breeding. The inbred lines of the breeding program of the 1980s entered in the Genebank as part of the maize collection.

Supplemental Figures:

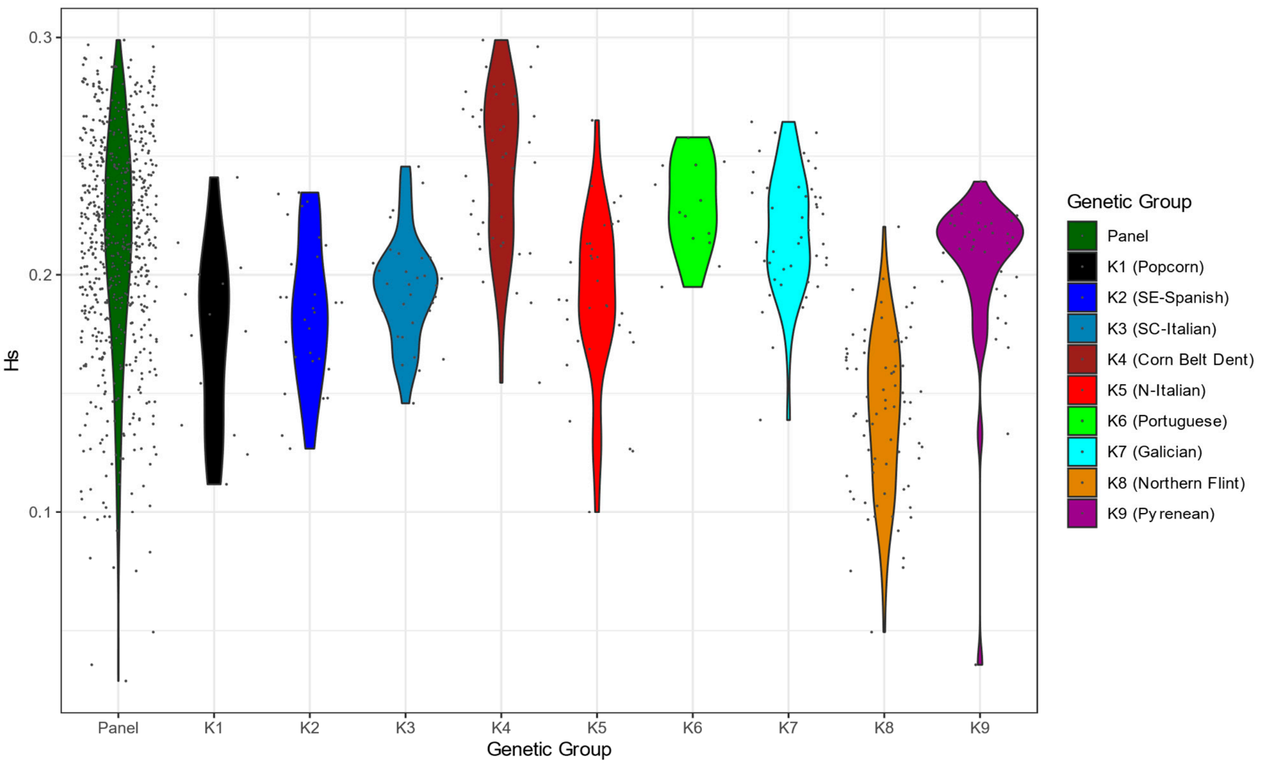

Figure S1: Expected heterozygosity (H_s) frequency for the complete panel and genetic groups from ADMIXTURE (K=9). Individual values for each landrace are represented with small dots.

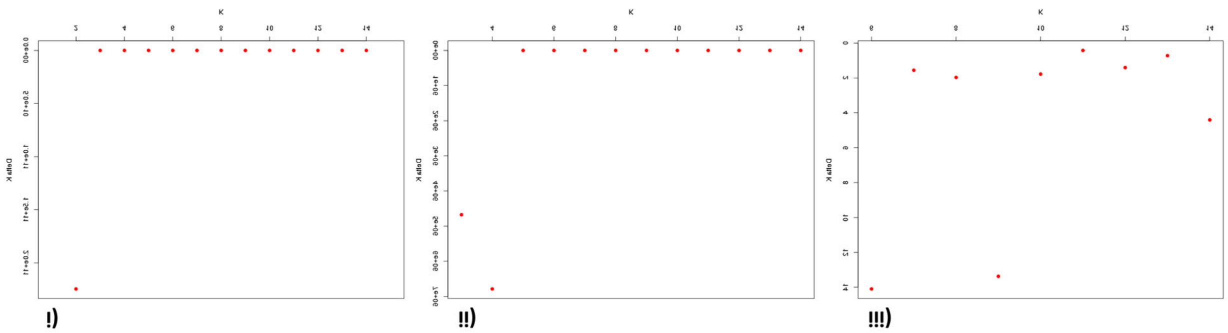

Figure S2: Determination of the K value for ADMIXTURE using deltaK calculated as per Evanno et al. (2005), representing i) K=2 to K=14, ii) K=3 to K=14, and iii) K=6 to K=14.

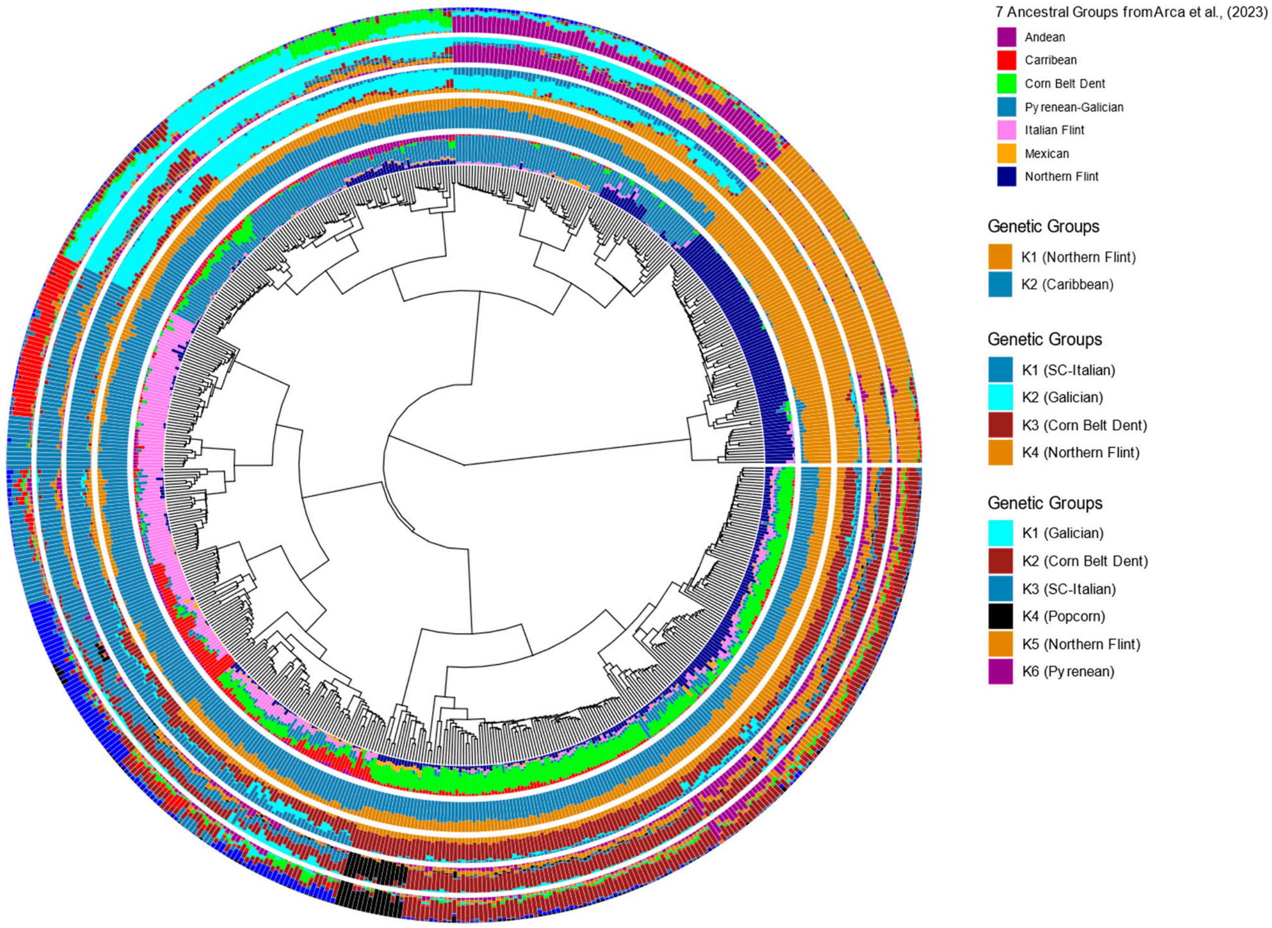

Figure S3: Ward dendrogram of genetic diversity based on Modified Rogers Distance of 626 landraces, for different K values. The rings are annotated as follows (from inside to external layer): i) barplots from penalized regression to 7 ancestral groups from Arca et al., (2023), ii) barplots from ADMIXTURE analysis (K=2), iii) barplots from ADMIXTURE analysis (K=4), iv) barplots from ADMIXTURE analysis (K=6), v) barplots from ADMIXTURE analysis (K=9).

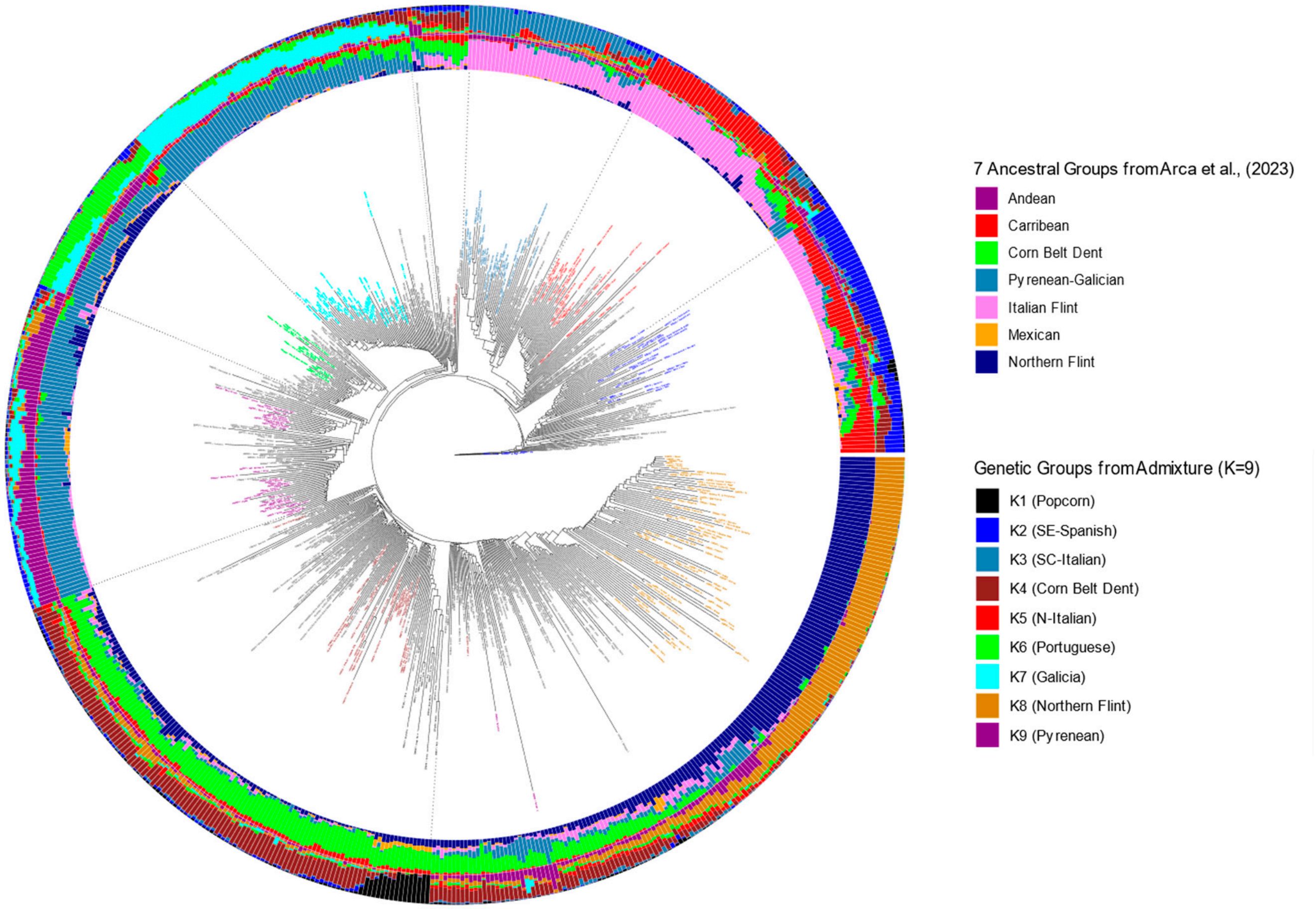

FigureS4: Neighbour joining dendrogram based on F_{st} between 626 landraces annotated with (from inside to external layer): i) barplots from penalized regression to 7 ancestral groups from Arca et al., (2023), ii) barplots from ADMIXTURE analysis (K=9). Label color of landrace code and common name corresponds to assignment to genetic groups of ADMIXTURE (K=9).

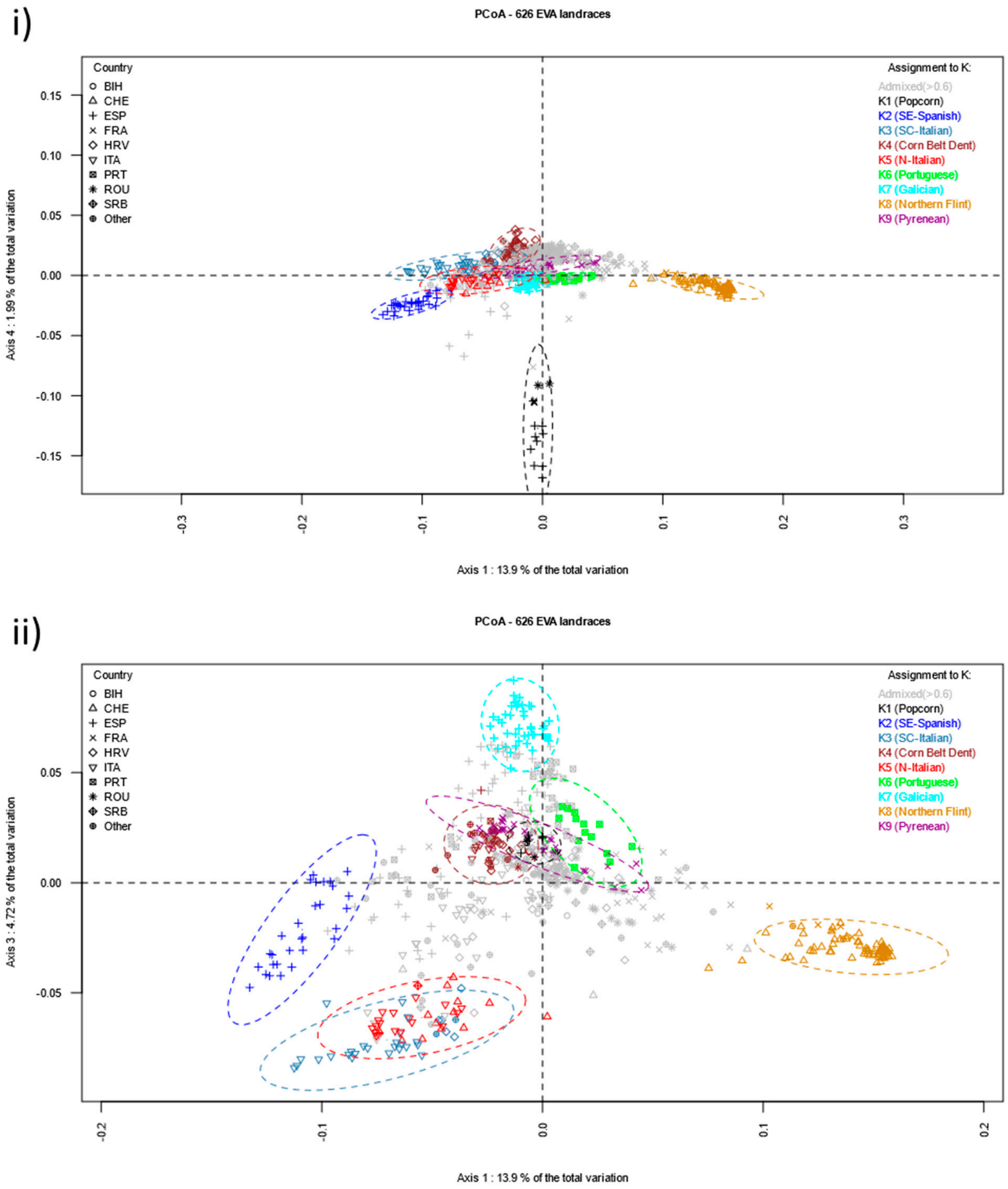

Figure S5: Principal Coordinate Analysis based on Modified Roger's Distance estimated with 23,412 SNP, representing i) axis 1 and axis 3, ii) axis 1 and axis 4. Different colors represent landraces assigned (>0.6) to one of 9 genetic groups of ADMIXTURE. Symbols represent the country of origin of each landrace (see legend).

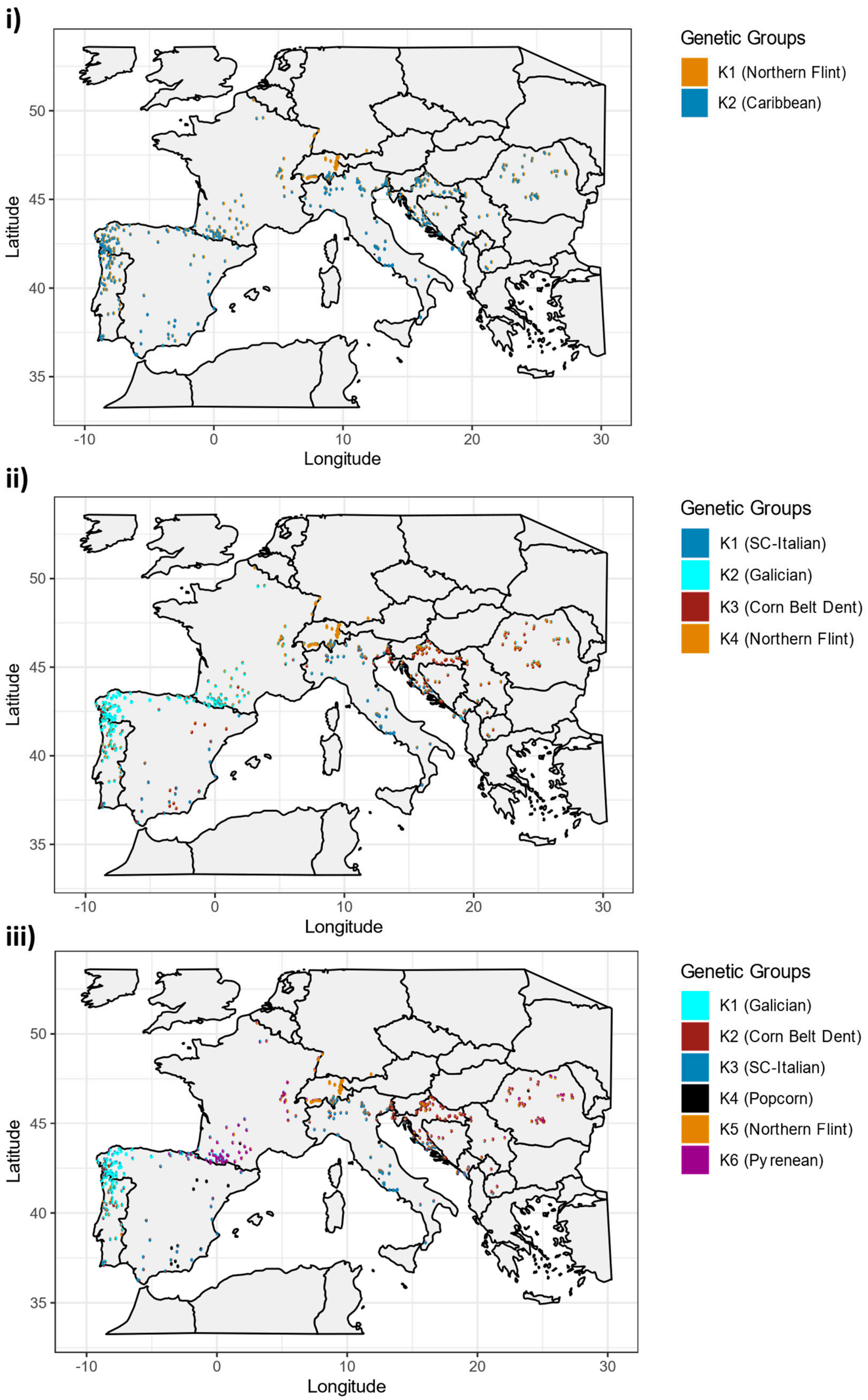

Figure S6: Map of origins of 483 EVA Maize accessions colored according to their belonging to genetic groups. Each accession is represented by a pie chart where the color composition reflects the proportion of genome ancestry to genetic groups of ADMIXTURE analysis (see legend) for i) K=2, ii) K=4, iii) K=6.

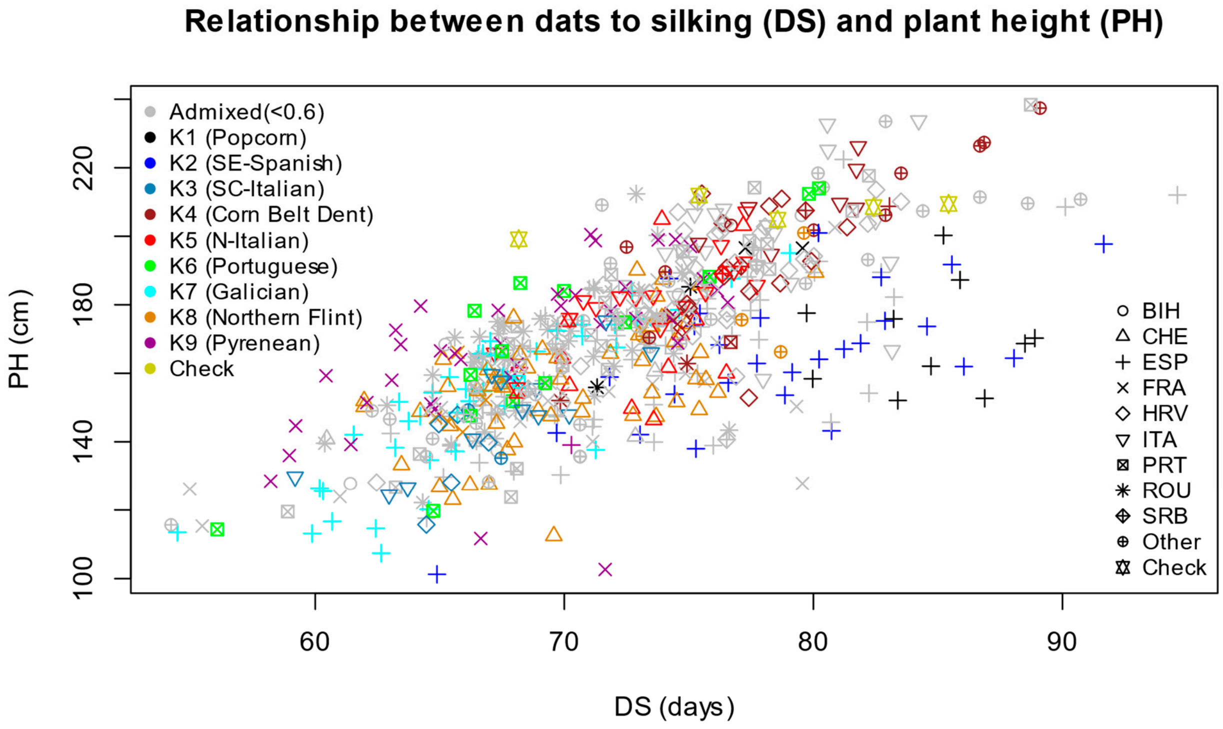

Figure S7: Relationship between female flowering (days to silking, DS) and plant height (PH) among 588 maize landraces of the EVA collection. Data is based on BLUEs from 2-9 trials per accession. Different symbols represent country of origin, while color represents genetic structure of landraces. Hybrid checks are represented with a different symbol and color.
